# Endoscopic Ultrasound (EUS)-Based Multimodal Diagnosis of a Rare Intramural Esophageal Squamous Cell Carcinoma: Case Report and Literature Review

**DOI:** 10.3390/jcm14238292

**Published:** 2025-11-21

**Authors:** Jacopo Fanizza, Francesco Vito Mandarino, Alberto Barchi, Gabriele Altieri, Riccardo Rosati, Ugo Elmore, Silvia Battaglia, Antonio Facciorusso, Lorenzo Fuccio, Gianfranco Donatelli, Daniela Finocchiaro, Maurilio Ponzoni, Silvio Danese, Giuseppe Dell’Anna

**Affiliations:** 1 Gastroenterology and Gastrointestinal Endoscopy Division, IRCCS San Raffaele Hospital, Via Olgettina 60, 20132 Milan, Italy; fanizza.jacopo@hsr.it (J.F.); mandarino.francesco@hsr.it (F.V.M.); barchi.alberto@hsr.it (A.B.); altieri.gabriele@hsr.it (G.A.); sdanese@hotmail.com (S.D.); 2 Faculty of Medicine and Surgery, Vita-Salute San Raffaele University, Via Olgettina 56, 20132 Milan, Italy; rosati.riccardo@hsr.it (R.R.); elmore.ugo@hsr.it (U.E.); battaglia.silvia@hsr.it (S.B.); ponzoni.maurilio@hsr.it (M.P.); 3 Gastrointestinal Surgery Unit, IRCCS San Raffaele Hospital, Via Olgettina 60, 20132 Milan, Italy; 4 Gastroenterology Unit, Faculty of Medicine and Surgery, University of Salento, 73100 Lecce, Italy; antonio.facciorusso@virgilio.it; 5 Gastroenterology Unit, Department of Medical and Surgical Sciences, IRCCS Azienda Ospedaliero-Universitaria di Bologna (IRCCS University Hospital of Bologna), University of Bologna, 40125 Bologna, Italy; lorenzo.fuccio3@unibo.it; 6 Unite d’Endoscopie Interventionnelle, Hôpital Prive des Peupliers, 75013 Paris, France; donatelligianfranco@gmail.com; 7 Department of Clinical Medicine and Surgery, University of Naples “Federico II”, 80126 Naples, Italy; 8 Department of Pathology, Division of Experimental Oncology, IRCCS San Raffaele Scientific Institute, 20132 Milan, Italy; finocchiaro.daniela@hsr.it; 9 Gastroenterology and Gastrointestinal Endoscopy Division, IRCCS Policlinico San Donato, Piazza Edmondo Malan 2, 20097 San Donato Milanese, Italy

**Keywords:** esophageal squamous cell carcinoma, Endoscopic Ultrasound (EUS), intramural, FNB, PET

## Abstract

Esophageal squamous cell carcinoma (ESCC) is the most prevalent histological subtype of esophageal cancer worldwide, typically manifesting as an endoluminal mass with overt mucosal involvement. Exceptionally, however, ESCC may present with an intramural growth pattern beneath an apparently intact mucosal surface, a presentation that is exceedingly rare and prone to misdiagnosis. In such cases, repeated endoscopic biopsies are frequently non-diagnostic, thereby delaying appropriate management. Endoscopic ultrasound (EUS) has emerged as the cornerstone for detecting and characterizing intramural lesions, enabling assessment of tumor infiltration depth and nodal status, while complementary imaging with CT and PET contributes to accurate staging. To date, only a handful of intramural ESCC cases have been described, and their clinical, endoscopic, and radiological features remain poorly delineated. This review appraises the existing literature on primary intramural ESCC with intact mucosa, with the dual aims of summarizing the diagnostic challenges and highlighting the value of a multimodal approach to avoid unnecessary surgical interventions. Furthermore, we report an additional case from our experience, which underscores the critical role of EUS and integrated imaging in achieving timely and accurate diagnosis of this unusual entity.

## 1. Introduction

Esophageal cancer remains a major global health burden, representing the eighth most frequently diagnosed cancer and the sixth leading cause of cancer-related death, with annual estimates exceeding 600,000 new cases and 540,000 deaths [1]. The overwhelming proportion of esophageal cancers are carcinomas, primarily divided into two histological subtypes: squamous cell carcinoma (ESCC), originating from the squamous epithelium throughout the esophagus, and adenocarcinoma, which arises almost exclusively in the distal third, typically in the context of Barrett’s esophagus [2,3]. ESCC represents approximately 88–90% of all esophageal carcinomas worldwide and remains the predominant form in Asian, African, and Eastern European countries [4]. Major risk factors for ESCC include heavy alcohol use and smoking. Other contributors are nutritional deficiencies, nitrosamine exposure, achalasia, poor oral hygiene, and frequent intake of very hot foods or drinks [5]. In most cases, ESCC is diagnosed at an advanced stage, when patients are already symptomatic, most often with progressive dysphagia. Endoscopically, it usually presents as a polypoid, exophytic, or ulcerative mass causing luminal stenosis, with mucosal involvement that generally allows confirmation by biopsy [6]. In contrast, esophageal tumors of mesenchymal origin (e.g., leiomyomas, lipomas, or melanomas) typically demonstrate submucosal growth beneath intact mucosa and are most often benign [7]. Endoscopic ultrasound (EUS) plays a pivotal role in the staging of esophageal cancer and in establishing a differential diagnosis of submucosal lesions. It is the most accurate modality for locoregional assessment, providing detailed evaluation of tumor invasion depth and regional lymph node involvement [8]. By differentiating early-stage lesions suitable for endoscopic resection (T1a) from those requiring surgery or neoadjuvant therapy (advanced T1b, T2–T4), EUS directly guides therapeutic decision-making. Furthermore, its ability to perform fine-needle aspiration (FNA) enhances diagnostic precision and complements CT and PET in comprehensive staging. Despite limitations such as difficulty traversing tight strictures and operator dependency, EUS remains an indispensable tool for prognosis and multidisciplinary management of esophageal carcinoma [9]. A completely intramural growth pattern of primary ESCC, with an apparently intact mucosal surface and absence of typical endoluminal changes, represents an exceedingly rare occurrence. To date, only a handful of such cases have been reported. In these cases, repeated mucosal biopsies are often negative, making the diagnosis particularly challenging and increasing the risk of delay or misdiagnosis. EUS has proven to be a crucial tool, as it enables assessment of intramural invasion and provides diagnostic confirmation; however, a truly accurate clinical assessment requires a multimodal approach, in which PET and CT also play an indispensable role in the precise characterization and staging of these lesions. Given the exceptional rarity of primary ESCC presenting an entirely intramural growth pattern, this entity remains poorly characterized and often underrecognized in clinical practice. Its atypical presentation poses significant diagnostic challenges, particularly because repeated endoscopic biopsies may fail to provide histological confirmation, thereby increasing the risk of diagnostic delay or misinterpretation. For this reason, a systematic appraisal of the available literature is warranted in order to better delineate its clinical, endoscopic, and radiologic features, as well as to highlight the diagnostic strategies most effective in this context to avoid unnecessary surgical procedures, which may be associated with significant complications [10,11], particularly in the setting of metastatic disease. The aim of the present review is therefore to collect and analyze all cases of intramural ESCC with intact mucosa reported to date, in order to provide a comprehensive overview of this unusual presentation. Moreover, we report our own case, which adds to the limited body of evidence and underscores the importance of a multimodal diagnostic approach in ensuring timely and accurate diagnosis.

## 2. Materials and Methods

We conducted a systematic literature review to identify all published cases of primary ESCC with a completely intramural growth pattern and an apparently intact mucosal surface, with particular focus on the role of EUS in lesion diagnosis and staging. The search was performed across major electronic databases, including PubMed, Scopus, and Web of Science, using a combination of keywords such as “intramural esophageal squamous cell carcinoma,” “submucosal ESCC,” “intact mucosa,” “endoscopic ultrasound,” and related terms. Articles in English were included, with no temporal restrictions, provided they reported clinical, endoscopic, radiologic, or histologic data, with evidence of EUS evaluation of the lesion. Studies were excluded if mucosal growth was evident or if sufficient information on intramural localization or the use of EUS was lacking.

## 3. Clinical Case

**Figure 1:**
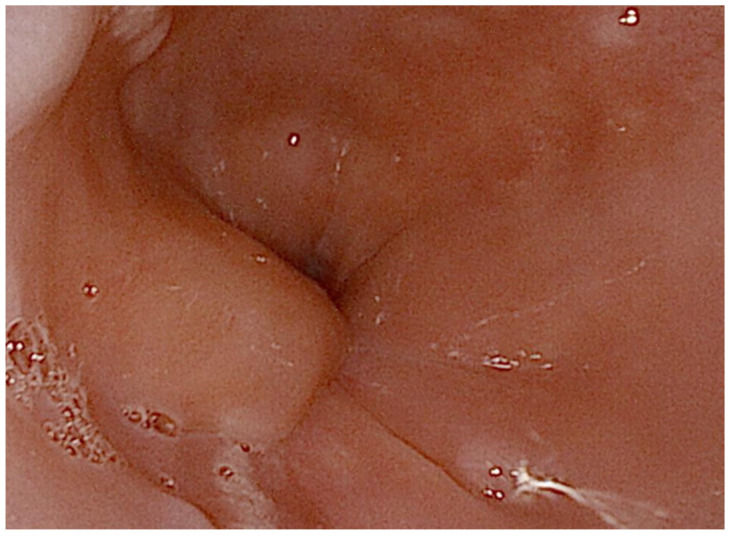
Upper gastrointestinal endoscopy demonstrated a bulging lesion in the lower esophagus, with the overlying mucosa appearing normal. The copyright of this figure belongs to the authors.

A 72-year-old male presented with progressively worsening dysphagia, primarily for solids, over five months, associated with significant weight loss and decreased appetite. He denied any history of heartburn, regurgitation, caustic ingestion, trauma, fever, cough, hemoptysis, voice changes, dyspnea, hematemesis, or melena. His past medical history was significant for bronchial asthma, allergic rhinitis, type 2 mellitus diabetes, obstructive sleep apnea on CPAP therapy, hypertension, dyslipidemia and benign prostatic hyperplasia. On physical examination, the patient was hemodynamically stable, with mild pallor noted; no lymphadenopathy was detected. Respiratory and gastrointestinal examinations were unremarkable. Routine laboratory investigations revealed a hemoglobin level of 12.1 g/dL, a total leukocyte count of 7800/µL, a platelet count of 210,000/µL, and normal serum creatinine and transaminase. Upper gastrointestinal endoscopy revealed a submucosal bulging lesion at 37 cm from the dental arcades, lined by intact squamous mucosa without ulcerations or erosions (Figure 1). The gastroesophageal junction was normal. Gastric biopsies showed chronic gastritis with Helicobacter pylori infection, but esophageal mucosal biopsies were unremarkable, raising suspicion of a submucosal tumor.

**Figure 2:**
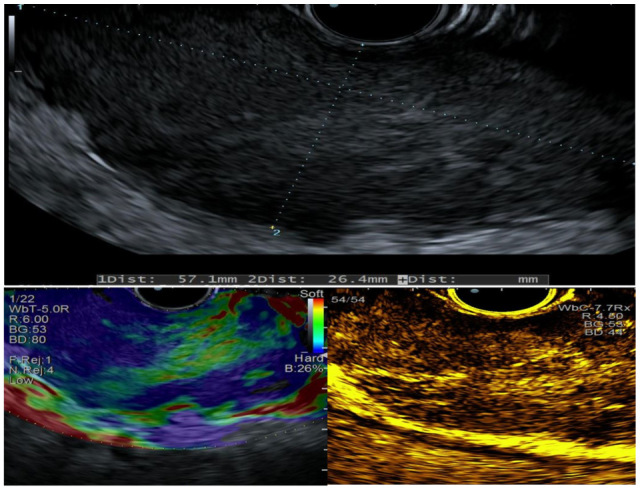
EUS characteristics evaluated at baseline, with elastography, and after contrast-enhanced imaging. The copyright of this figure belongs to the authors.

EUS revealed circumferential hypoechoic thickening of the esophageal wall involving the submucosa and muscularis propria (60 × 30 mm in size), with hard consistency on elastography and hypoenhancement following SonoVue (Bracco) injection (Figure 2).

**Figure 3:**
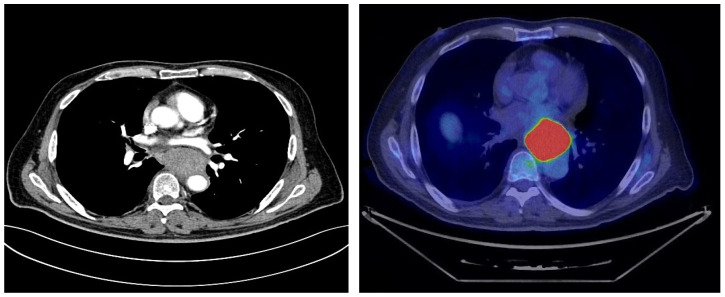
Cross-sectional CT imaging and FDG-PET demonstrated a thoracic mass in the absence of metastatic disease, but the exact site of origin remained unclear. The copyright of this figure belongs to the authors.

Cross-sectional imaging with computed tomography (CT) scans confirmed the presence of a retroesophageal mass with heterogeneous enhancement, but the differential diagnosis remained broad, including leiomyosarcoma, gastrointestinal stromal tumor, or atypical lymph node involvement (Figure 3). No distant metastases were detected. FDG-PET further characterized the lesion, showing intense tracer uptake confined to the retroesophageal mass, without other significant sites of pathological activity (Figure 3).

**Figure 4:**
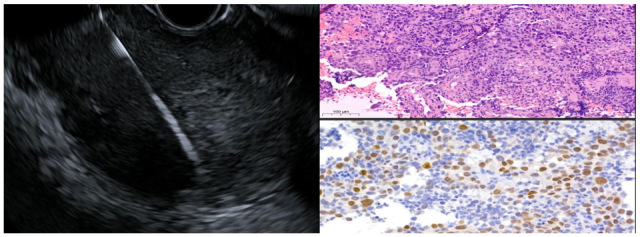
EUS-FNB with corresponding histopathological and immunohistochemical images of the obtained tissue. The copyright of this figure belongs to the authors.

EUS-guided fine-needle biopsy (FNB) using a 22-gauge fork-tip needle (SharkCore Covidien Medtronic, Minneapolis, MN, USA) was performed (Figure 4), supported by immunohistochemistry, which finally revealed the true nature of the lesion: a poorly differentiated ESCC G3 with an entirely intramural growth pattern (p-63+, CD-5-, CD-117-, TTF-1-, CDX-2-, Ki-67: 80%) (Figure 4).

Given the unusual growth pattern under intact mucosa and histological confirmation of ESCC, the case was referred to a multidisciplinary tumor board. Diagnostic laparoscopy with peritoneal washing and placement of a feeding jejunostomy was performed to optimize nutritional status and exclude occult peritoneal disease. The patient is currently under close multidisciplinary follow-up and awaits initiation of neoadjuvant chemoradiotherapy as part of the planned multimodal treatment strategy.

## 4. Results of the Literature Review

A total of thirteen case reports were identified through the database search. The main clinical characteristics of these patients are summarized in Table 1.

Submucosal or intramural ESCC represents an exceedingly rare clinicopathological entity that diverges sharply from the conventional growth pattern of classical esophageal squamous tumors. Whereas the vast majority of ESCCs originate from mucosal dysplasia and manifest endoscopically as ulcerated, exophytic, or polypoid masses readily amenable to biopsy, the intramural variant develops entirely beneath an intact squamous lining. This peculiar growth pattern poses significant diagnostic difficulties, as repeated mucosal biopsies often return negative, thereby delaying recognition and appropriate management. A careful appraisal of the literature reveals only a very limited number of well-documented cases, each contributing incremental insight into the clinical and pathological spectrum of this unusual disease presentation. The first case was reported by McGregor et al. in 1976, where autopsy findings suggested that carcinoma cells might have originated from squamous epithelium of intramural cysts located within the muscularis and deep submucosa [12]. Subsequent report by von Rahden et al., confirmed that this intramural growth pattern could be diagnosed only postoperatively, as standard mucosal biopsies consistently failed to detect malignant tissue [14]. Later, Sonthalia et al. emphasized the indispensable role of EUS-FNA, which in their case provided the first definitive diagnosis [15]. Kishino et al. highlighted the aggressive potential of this entity by describing a tumor with gastric and hepatic metastases [13], while Zhu et al. documented two cases successfully managed ESD, thus demonstrating the therapeutic and diagnostic utility of advanced endoscopic techniques [18]. More recently, Jia et al. reported a case of submucosal ESCC with synchronous lymph node metastasis, in which combined ESD and chemoradiotherapy led to regression of nodal disease, reinforcing the hypothesis that submucosal localization may predispose to early lymphatic spread due to the rich vascular and lymphatic network in the esophageal wall [21]. An additional point of discussion emerging from the literature concerns the uncertain histogenetic origin of these tumors. Two main hypotheses have been advanced [15]. The first proposes that submucosal ESCC may arise from esophageal cysts, diverticula, or from squamous metaplasia of esophageal glands: in this model, initial glandular hyperplasia progresses through metaplasia, dysplasia, carcinoma in situ, and finally invasive carcinoma [15]. The second hypothesis suggests that the tumor originates from pre-existing squamous epithelial cells that, through ductal structures, extend into the submucosa, where they acquire the capacity to proliferate beneath an otherwise intact mucosa. Although neither theory has been definitively proven, both highlight the potential for atypical biological behavior and may explain why these lesions often mimic benign subepithelial tumors in their earliest stages [15]. From a clinical standpoint, most reported patients presented with dysphagia, although incidental diagnoses have also been described. Tumor localization spans the entire esophagus, with lesion size ranging from small nodules of less than 2 cm to bulky masses exceeding 5 cm. Prognosis appears heterogeneous: while some cases showed rapid progression and early mortality, others demonstrated long-term disease control when diagnosed early and managed with multimodal strategies, particularly when ESD was combined with adjuvant chemoradiotherapy. Taken together, the cumulative evidence, comprising 13 cases worldwide, underscores that intramural ESCC is a highly heterogeneous disease, both in clinical presentation and biological behavior. Several recurring themes can be identified: standard endoscopic biopsies are frequently non-diagnostic; EUS, with or without FNB, remains the cornerstone for accurate diagnosis; ESD serves as both a curative and staging modality; and multimodal treatment appears to improve long-term outcomes, particularly in the presence of lymph node metastasis. Finally, awareness of this entity is critical, as its endoscopic resemblance to benign subepithelial lesions risks underdiagnosis and therapeutic delay.

## 5. Discussion

Intramural growth of ESCC is an exceedingly rare clinical entity, with only a handful of cases documented in the literature. In contrast to conventional ESCC, which typically presents as a mucosal lesion with ulceration, polypoid formation, or luminal narrowing, the intramural variant predominantly develops within the esophageal wall while preserving the overlying mucosa. This atypical growth pattern poses significant diagnostic challenges, as standard mucosal biopsies frequently fail to capture malignant tissue, often yielding negative or non-diagnostic results and resulting in substantial delays in diagnosis and management. We report a case of a 72-year-old male presenting with progressive dysphagia, weight loss, and anorexia, symptoms characteristic of advanced esophageal malignancy. Endoscopic evaluation, however, revealed a submucosal bulge with intact overlying mucosa, resembling benign subepithelial lesions such as leiomyoma or gastrointestinal stromal tumor (GIST). Multiple mucosal biopsies were inconclusive, highlighting the limitations of conventional endoscopic sampling in cases of intramural tumor growth. EUS proved decisive in this case. It accurately delineated the submucosal origin and extent of the lesion and excluded a mucosal source, thereby refining the differential diagnosis. Furthermore, EUS-FNB provided adequate tissue for histopathological and immunohistochemical analysis, ultimately establishing the diagnosis of poorly differentiated ESCC with complete intramural growth. Previous reports consistently emphasize the central role of EUS in such scenarios, as patients with intramural ESCC typically present with dysphagia and other non-specific gastrointestinal symptoms. Repeated negative mucosal biopsies often contribute to diagnostic delays, whereas EUS enables precise lesion localization, assessment of size and extent, and targeted tissue acquisition via EUS-FNA or FNB. These findings underscore EUS as the diagnostic modality of choice for submucosal esophageal lesions with intact mucosa and inconclusive biopsy results. Beyond its critical role in rare intramural variants, EUS remains the reference standard for loco-regional staging of esophageal carcinoma. Its high-resolution imaging of the esophageal wall, combined with tissue sampling capabilities, allows for accurate assessment of tumor invasion, achieving overall accuracy exceeding 80%, with values surpassing 90% for T3 tumors [24], and outperforming CT, particularly in early-stage disease [25]. Meta-analytic data report sensitivity and specificity of 81.6% and 99.4% for T1, 81.4% and 96.3% for T2, 91.4% and 94.4% for T3, and 92.4% and 97.4% for T4 tumors, respectively [26]. A critical clinical challenge is differentiating T1a (mucosa-confined) from T1b (submucosal invasion), given the substantial differences in lymph node metastasis risk, which ranges from 3–6% in T1a/T1b-SM1 to 21–24% in T1b-SM2/SM3 [24]. Despite technological advances, EUS alone has limited accuracy for this distinction, necessitating complementary techniques such as endoscopic mucosal resection (EMR) or ESD for definitive histopathological staging. The advent of high-frequency miniprobes has enhanced resolution and diagnostic accuracy in early-stage disease, allowing improved discrimination between T1 and T2 lesions, with reported accuracy up to 92% [27]. EUS is equally important for nodal staging. Morphological criteria alone—such as hypoechogenicity, round shape, smooth borders, and size >1 cm—offer moderate sensitivity. When combined with EUS-FNA, diagnostic accuracy substantially increases, with sensitivity approaching 96% and overall accuracy exceeding 90%, particularly for mediastinal and celiac nodes. In contrast, EUS has a more limited role in detecting distant metastases, emphasizing the need for complementary imaging modalities such as CT and PET [25]. The clinical impact of EUS is profound: accurately staged T1a tumors may be managed endoscopically, whereas T1b-SM2/SM3 and T2 tumors typically warrant multidisciplinary surgical evaluation, and locally advanced disease (T3–T4 or N+) is best managed with neoadjuvant therapy [28]. In addition to cancer staging, EUS is invaluable in the assessment of esophageal subepithelial lesions (SELs), many of which are incidentally detected during routine endoscopy. EUS provides precise characterization by identifying the layer of origin, echogenicity, margins, and vascular patterns, offering important diagnostic and prognostic insights. Its accuracy in predicting malignant potential demonstrates a sensitivity of 64% and specificity of 80%, which can be significantly improved with advanced modalities such as elastography and contrast-enhanced EUS, capable of differentiating leiomyomas from GISTs with over 95% accuracy [29]. Both techniques provide complementary functional information—vascularity and tissue stiffness—that enhance diagnostic accuracy in differentiating benign from malignant gastrointestinal lesions, including subepithelial and intramural tumors such as rare intramural variants of ESCC [30]. EUS elastography assesses tissue stiffness by measuring deformation (strain) in response to mechanical compression. Neoplastic or fibrotic tissues generally exhibit increased stiffness compared with benign or cystic lesions. Qualitative elastography generates color maps, where blue indicates harder tissue and green or red softer tissue, while the semi-quantitative approach provides a strain ratio comparing the lesion with a reference area [31]. Several studies have shown that potentially malignant subepithelial tumors present significantly higher strain ratios (mean 8–9) than leiomyomas or other benign lesions [31]. When combined with conventional EUS features, such as hypoechogenicity, irregular margins, and internal heterogeneity, elastography improves overall diagnostic performance, with reported accuracy exceeding 85% [32]. In esophageal pathology, a stiffer elastographic pattern may raise suspicion of infiltrating carcinoma even in the absence of mucosal disruption, as observed in intramural ESCC. However, the technique remains limited by operator dependency, artifacts, and lack of inter-system standardization. For these reasons, the European Society of Gastrointestinal Endoscopy (ESGE) guidelines recommend EUS-E as a complementary tool, but not as a stand-alone diagnostic modality [33]. Contrast-enhanced EUS (CE-EUS), using intravenous microbubble agents enables real-time evaluation of intralesional perfusion and visualization of the microvascular architecture, allowing differentiation between viable and necrotic or cystic areas [34]. CE-EUS improves the yield of EUS-FNB by targeting the most viable and vascularized regions. Recent meta-analyses have reported a sensitivity of 90–96% and a specificity of 65–70% for CE-EUS in distinguishing malignant from benign subepithelial lesions [35]. In esophageal malignancies, CE-EUS can also differentiate hypoperfused fibrotic areas from hyperperfused viable tumor tissue, thus improving T-staging accuracy and enabling assessment of response to neoadjuvant therapy [35]. Overall, the combination of EUS-E and CE-EUS marks the transition from a purely structural to a functional endosonographic approach, enabling non-invasive assessment of the biomechanical and vascular characteristics of gastrointestinal wall lesions. When applied to subepithelial and intramural tumors, these techniques refine differential diagnosis, guide tissue sampling, and enhance risk stratification. In complex or rare conditions—such as intramural esophageal squamous cell carcinoma—the presence of a hard, hypoperfused lesion arising from the submucosa or muscularis propria should raise suspicion of malignancy and prompt timely tissue acquisition. Tissue acquisition techniques further enhance diagnostic yield: EUS-FNA and FNB achieve results ranging from 46% to 100%, with higher yields in lesions larger than 4 cm, and FNB providing superior tissue architecture preservation. These capabilities position EUS as a cornerstone not only for diagnosis but also for risk stratification and therapeutic planning, guiding decisions from surveillance to surgical or endoscopic resection [36]. Collectively, these data highlight the indispensable role of EUS across the spectrum of esophageal pathology. Looking forward, the integration of these modalities with artificial intelligence algorithms and radiomic analysis may further standardize interpretation and improve diagnostic precision in complex gastrointestinal neoplasms [37]. In rare intramural ESCC, clinicians should maintain a high index of suspicion when encountering persistent dysphagia in patients with apparently submucosal esophageal lesions, particularly in elderly individuals or high-risk populations. Standard mucosal biopsies are often insufficient, making EUS-FNA or FNB essential for accurate preoperative diagnosis. Recognizing the intramural growth pattern is crucial to avoid unnecessary surgical interventions and to enable timely initiation of appropriate multimodal therapy, particularly in the presence of metastatic disease. Despite the preservation of the overlying mucosa, intramural ESCC may exhibit aggressive behavior and metastatic potential, underscoring the need for careful staging and close follow-up.

## 6. Conclusions

Intramural ESCC represents an exceptionally rare entity that poses significant diagnostic and therapeutic challenges. The absence of mucosal involvement often results in repeatedly negative endoscopic biopsies, with a consequent risk of delayed diagnosis and treatment. This underscores the importance of maintaining a high index of suspicion in cases where endoscopic evaluation fails to demonstrate malignancy, but the clinical and radiological findings remain strongly suggestive. In this setting, EUS plays a pivotal role. This technique not only allows precise characterization of the intramural nature of the lesion and its relationship to the esophageal wall layers but also provides cytological samples in a minimally invasive and safe manner. Such an approach can reduce the need for surgical exploration undertaken solely for diagnostic purposes and facilitates earlier initiation of appropriate treatment. Clinically, these neoplasms may present typical symptoms of esophageal cancer, such as dysphagia, while endoscopic appearance may mimic benign subepithelial lesions, adding to the diagnostic complexity. For this reason, it is crucial that gastroenterologists, endoscopists, oncologists, and surgeons are aware of this rare presentation and consider it in the differential diagnosis of intramural esophageal masses. In conclusion, although rare, intramural ESCC should be suspected when clinical and imaging findings suggest malignancy in the absence of histological confirmation. The use of advanced diagnostic modalities, particularly EUS and PET, is essential to achieve an early and accurate diagnosis, guide the most appropriate therapeutic strategy, and avoid unnecessary delays or overly invasive diagnostic procedures.

## 7. Limitations & Future Directions

The present report adds to the limited body of evidence on intramural ESCC but must be interpreted considering several limitations. First, both our case and the previously published reports are anecdotal in nature, precluding robust conclusions on incidence, natural history, and optimal management. The heterogeneity of clinical presentations, diagnostic modalities, and therapeutic strategies across cases further limits the ability to draw standardized recommendations. In addition, the rarity of this presentation increases the likelihood of delayed recognition and potential underreporting, given that lesions may be misclassified as benign submucosal tumors. Future efforts should focus on multicenter case series and prospective registries to systematically collect clinical, endoscopic, radiologic, and histopathologic data on intramural ESCC. Such collaborative work would enable a more accurate characterization of its biological behavior, refine diagnostic algorithms, and identify prognostic factors. Moreover, advances in EUS technology—including elastography, contrast enhancement, and novel needle designs—should be prospectively validated in this setting to optimize tissue acquisition and staging accuracy. Ultimately, only through larger, coordinated investigations will it be possible to establish evidence-based guidelines for the diagnosis, risk stratification, and management of this exceptionally rare form of ESCC.

## Figures and Tables

**Table 1:** Review of case reports of esophageal intramural squamous cell carcinomas. RT, radiotherapy; CRT, chemoradiotherapy; ESD, endoscopic submucosal dissection.

Authors & Year	Gender & Age	Symptoms	Tumor Size (mm)	Localization	Metastasis	Initial Treatment	Outcomes
D. H. Mc Gregor et al., 1976 [12]	Male, 65	Dysphagia	-	Proximal esophagus	None	RT	Death
T. Kishino et al., 2000 [13]	Male, 60	Dysphagia	-	Distal esophagus	Gastric and liver	Surgery	Death
B. H. A. von Rahden et al., 2006 [14]	Male, 58	Dysphagia	-	Proximal esophagus	None	Surgery + CRT	No recurrence or metastasis
N. Sonthalia et al., 2016 [15]	Female, 45	Dysphagia	33 × 25	Distal esophagus	Bone	CRT	After 2 months, dysphagia partially relieved; no change in size
N. S. Choudhary et al., 2016 [16]	Male, 74	Dysphagia	18 × 15	Proximal-middle esophagus	None	Unspecified	Unspecified
R. M. Shanmugam et al., 2019 [17]	Male, 50	Dysphagia	29 × 29	Middle esophagus	Lymph nodes, lung	Unspecified	Unspecified
H. Zhu et al., 2020 [18]	Male, 63	Abdominal distension	-	Proximal esophagus	None	ESD + postoperative RT for positive margin	No recurrence or metastasis
H. Zhu et al., 2020 [18]	Male, 65	Dysphagia	15 × 15 × 10	Middle esophagus	None	ESD	No recurrence or metastasis
Hanghai Pan et al., 2020 [19]	Female, 78	Dysphagia, nausea, vomiting	-	Middle and distal esophagus	None	Unspecified	Unspecified
W. Wang et al., 2021 [20]	Male, 58	No	5.7 × 2.2	Middle esophagus	None	ESD than surgery for positive margin	No recurrence or metastasis
Y. Jia et al., 2022 [21]	Female, 63	No	15	Proximal esophagus	Lymph nodes	ESD + CRT	No recurrence; regressing lymph node metastases
L. Yang et al., 2023 [22]	Male, 48	Dysphagia	17 × 9.4	Proximal esophagus	No	Enucleation + CT	No recurrence or metastasis
J. Qiu et al., 2025 [23]	Female, 51	Dysphagia	-	NA	No	ESD + CRT	No recurrence or metastasis

## Data Availability

Data sharing does not apply to this article as no new data were created or analyzed in this study.
